# Effects of liming and different land use types on phosphorus sorption characteristics in acidic agricultural soil of Sodo Zuria Woreda, Southern Ethiopia

**DOI:** 10.1016/j.heliyon.2023.e14124

**Published:** 2023-02-26

**Authors:** Eshetu Yigezu, Fanuel Laekemariam, Alemayehu Kiflu

**Affiliations:** a Wolaita Zone Water, Irrigation and Mines Development Department, Ethiopia; b Wolaita Sodo University, College of Agriculture, Wolaita Sodo, Ethiopia; c Hawassa University, College of Agriculture, Hawassa, Ethiopia

**Keywords:** Adsorption maximum, SPR and Freundlich models

## Abstract

**Background:**

Phosphorus (P) sorption measurements and lime application are of great importance for the sustainable management of P-adsorptive soils.

**Aim:**

Therefore, this study aimed to evaluate soil phosphorus sorption under different land-use types and to assess lime input during phosphorus sorption. Treatments include the land use of enclosures, grazing, cultivated and ensut land.

**Method:**

Surface soil samples were collected to study the physicochemical properties of specific soils. Lime was mixed with the soils of cultivated land for 30 days. P sorption was subsequently assessed for all land-use types by equilibrating soil samples in 0.01 M CaCl_2_ containing 30 mL of KH_2_PO_4_ at 0, 10, 20, 30, 40, and 50 mg/L.

**Result:**

The results showed that land use type had a significant impact on external P demand (EPR). The Langmuir model proved useful information in explaining P sorption. P fixation ranged from 136 to 731.67 mg.kg^−1^ according to Langmuir model and EPR values ranged from 45.9 to 398.7 mg P kg^−1^ soil. Exclusory area soil has high sorption compared to other land-use whereas enset land-uses the lowest sorption. The result of correlation analysis revealed that exchangeable Aluminium and clay had positively correlated on P-sorption maximum and SPR of both models.

**Conclusion:**

It was concluded that there was a significant difference among land-use systems of P-sorption and there had an influence of lime on acidic soil to reduce EPR. So black market p-fertilizer utilization is not recommended to study site. Liming also restored the soil chemistry of cultivated land. Nevertheless, field trials were proposed to validate mineralization rates and EPR values in cultivated soils.

## 1 Introduction

Soil acidity is a critical issue requiring urgent attention in most highlands of Ethiopia because of its impact on crop production and productivity [1]. Most acidic soils have poor chemical and biological properties. In acid soils, phosphorus (P) is highly fixed due to intensive weathering and leaching attributed to high rainfall conditions [2]. One unique characteristic of P is its slow diffusion and high fixation in soils and, therefore it is imperative to apply P fertilizers for improving crop growth and yield [3]. Furthermore, the limited availability of P in soils may be attributed to severe P fixation or sorption. In acid soils, Al and Fe adsorb P and in neutral soils, P retention on Ca was dominated by precipitation reactions [3]. P fixation in acid soils can be corrected through liming. Lime raises soil pH to the level that is suitable for maximum nutrient availability [4]. Additionally, liming increases soil P, Ca, Mg and microbial activities [5].

The P sorption isotherm can be used to calculate the Standard Phosphorus Requirement (SPR) for most crops at an equilibrium concentration of P(0.2) mg L^−1^ soil solution. Langmuir and Freundlich’s models were the most widely used models to describe P-sorption characteristics of soils and draw external P-requirements [6]. Freundlich models were able to discriminate soils based on their ability to sorb/adsorb P from soil solutions. They also give an insight into the strength of P-adsorption on the surface of a particular soil.

In Sodo Zuria Woreda soil acidity and inadequate P fertilizer application were among the major challenges affecting crop productivity. Soil acidity in the area may arise from crop residue removal for firewood consumption. Farmers use Urea and DAP fertilizers for an extended period of time, an inappropriate way which might have contributed to soil acidity. Recommending fertilizer requirements of crops based on biological tests alone is unlikely to address the problem of plant nutrition unless used in combination with soil test results. Moreover, researchers stressed that soil testing is essential for accurate and profitable fertilizer recommendations provided that the soil test result correlates to the crop response [7]. There are many research articles concerning plant and soil nutrient availability. However, research articles and knowledge is lacking about the P sorption and lime application rate of different land use type in Sodo Zuria. Thus, the objectives of the study were to evaluate soil P sorption under different land-use types and to evaluate the influence of lime on P sorption.

## 2 Materials and methods

### 2.1 Description of study site

The study was conducted in Kuto Sorfela Kebele of Sodo Zuria woreda, Wolaita Zone of Southern Ethiopia. Geographically the woreda is situated at latitude 6°49′ 59.99 N and longitude 37° 44′ 59.59″ E. The average annual rainfall is 1200 mm per annual, while the daily temperature varies from 15° C to 30° C. Soils of the study area is grouped as Nitosols.

The study area, in general, comprises 3% flat, gentle 12%, undulating 23%, and steeping lands is 62%. The slope declines east to West with all drainage being directed to the Waja River. The agroecology of the study area is dominated by midland covers about 57% of the total area, and the remaining 43% is highland with rugged mountains and slopes.

### 2.2 Site selection, treatments, and experimental design

A preliminary reconnaissance survey on farms was conducted. Four land-use types were identified and purposively selected. These include enset fields (perineal crop fields with high organic fertilizer input); cultivated land (annual crop fields with inorganic fertilizer), enclosure areas, and open grazing land. Enset (*Ensete ventricosum*) field was selected as one of the land uses to represent organic soil because it is as old as agriculture in the study area and used as a staple food [8]. Moreover, cultivated land was selected as the second land use because it is the dominant land use and crop response to phosphorus fertilizer application is low, whereas exclusion area and grassland have been selected as the third and fourth land-use systems because they are the major land use types practiced in that Woreda. Purposive site selection method was implemented to obtain four land use types within the same topographic positions along the toposequence at Kuto Sorpela Kebeles. The topographic positions (upper, middle, and lower) were used as a block and land use systems were used as treatments. The experiment was designed and arranged in a randomized complete block design (RCBD). Thus, a total of 12 plots (4 land uses x 3 blocks) were identified for investigation. While evaluating the P sorption characteristics, lime-incubated soil from a cultivated field was also included as a treatment to recommend it for the local farmers.

### 2.3 Soil sampling and analysis

Surface soil samples at depth of 0–20 cm from each land use were collected using an auger. From each field, 10 sub-samples in a Zigzag pattern were taken to make 1 kg of composited soil sample. Following the standard procedure outlined in Sahlemedhin and Taye [9], soil samples were processed and analyzed for selected physical and chemical properties. After drying the soil samples to constant weights in an oven at 105 °C, the bulk density of the soil was determined using a core sampler. A portion of the disturbed soil sample was taken and sieved with a 0.5 mm diameter sieve for organic matter determinations.

Particle size distribution analysis was done by the hydrometer method [10]. Textural classes were determined by Marshall’s Triangular coordinate system. Soil pH was measured as described by procedure using a glass electrode pH meter with a ratio of 1:2.5 soils to water [10]. Organic carbon was determined using the chromate wet Oxidation method [11] and the soil organic matter was calculated by multiplying the percent value of organic carbon by the conversion factor 1.724 Sahlemedhin and Taye [10]. Available phosphorous was determined by extracts using the ascorbic acid molybdenum blue method of Olsen extraction as described by Olsen et al. [12]. Exchangeable bases (Ca, Mg, K, and Na) were determined after extracting the soil samples with ammonium acetate (1 N NH_4_OAc) at pH 7.0. Exchangeable Ca and Mg in the extracts were analyzed using atomic absorption spectrophotometer, while Na and K were analyzed by a flame photometer [13].

### 2.4 Lime requirement (LR) determination

The LR was determined using the Shoemaker, McLean, and Pratt (SMP) single buffer procedure [14]. This method was widely used in Ethiopia and southern Ethiopia because it is recommended for soils requiring more than one ton of lime per acre and soils with a pH of less than 5.8 [15]. The soil was equilibrated with pH 7.5 buffer solution whereby reserve H is brought into the solution, which results in depression of pH which will be made and interoperated in terms of the lime required to meet raise the pH to a desired value. Soil pH in the SMP buffer solution was found to be 5.1. SMP single-buffer procedure uses a regression equation to calculate the required lime for different target pH values. In this case, the target pH in water was 6.5 and it was calculated by (Equ. 1)

(1) LR (6*.*5) = 1*.*867(pHB)^2^ − 31*.*82 (pHB) + 131*.*23,

where LR (6.5) is lime requirement to bring soil pH to 6.5.

pHB is pH value of SMP buffer solution. Thus, based on the calculation result, 17.5 t/ha CaCO_3_ was used to bring the soil to the target pH (6.5 in water).

### 2.5 Soil incubation

Approximately 1 kg of cultivated soil composite sample was packed into a 2000 ml polythene bag and thoroughly mixed with equivalent weights of lime requirement treatments (0 and 100% lime rate). The samples were incubated for 30 days at roughly field water holding capacity to allow the lime to react with the soils before being air-dried for laboratory P sorption studies alongside unlimited samples.

### 2.6 P-sorption study

A stock solution of 0, 10, 20, 30, 40, and 50 mgl^−1^ P was tested on air-dried 2 g of incubated cultivated soil with lime and control (KH_2_PO_4_). Each P sorption set was repeated three times. The sorption rate of phosphorus was determined using batch equilibrium methods in which soil samples were agitated with P solutions of known concentrations [16]. Soil subsamples were taken from an incubated sample prepared for the study of P-sorption. Phosphorus (KH_2_PO_4_) was dissolved in distilled water in a 0.01 M solution of calcium chloride. To improve centrifugation and minimize cation exchange, the CaCl_2_ solution was used as the aqueous solvent phase [17].

According to the method of Fernandes and Coutinho [18] to study the sorption of P by soils, 2 g air-dried samples of each soil were placed in 100 ml plastic bottle to leave free space for with 25 ml of 0.01 M CaCl_2_ in which the final volume was adjusted to 30 ml. Continuous mixing was provided during the experimental period with a constant agitation speed of 350 rpm for better mass transfer with a high interfacial area of contact. Afterward, a calculated amount of stock solution of P for each rate was added.

The mixture was shaken at a maximum speed of 380 rpm for 30 min before being equilibrated for 24 h. The suspension was filtered through Whatman paper No. 42 filter paper after equilibration time, and the concentration of P in the clear extract was determined using the ascorbic acid method. Sorbed P was defined as phosphorus that had vanished from the solution and was plotted against the P concentration in the solution to obtain a P sorption isotherm. For each soil, a blank was run with the same amount of soil and a total volume of 30 ml 0.01 CaCl_2_ solution (without P) was added and the same procedure was followed. During the analysis, this serves as a background control to detect interfering compounds or contaminated soils.

The P sorption data for the soils was fitted into the following forms of Langmuir equation (Eq. (2)) because linear regression was convenient and the best data-fitting process and Freundlich equation (Eq. (4)).

#### 2.6.1 Langmuir equations

(2) C/X = 1/K.Xm + C/Xm

where.

C (mgl^−1^) was the equilibrium concentration,
X (mg kg^−1^) was the amount of P adsorbed per unit mass of adsorbent (Eq. (3)),
K (L mg^−1^) was a constant related to the energy of sorption, and
Xm (mg kg^−1^) was P sorption maximum.

(3) X was calculated as CoVo –CfVf/mass of soil (kg)

where.

Co was the initial concentration,
Cf is final concentration, V was the volume of the solution.

The linear form of Eq. (1) was obtained by plotting the equilibrium concentration of phosphate (C) against the amount of phosphate adsorbed (X) and the slope of the graph equals to 1/Xm and the intercept of the graph is equal to 1/K.Xm. But K was easily determined by dividing the slope by intercept.

(4) Freundlich equation: X = KC^b^or logX = logkf + blogC

where, K and b (b < 1) are constants, X (mgkg^−1^) is the amount of P adsorbed per unit mass of adsorbent, and C (mgl^−1^) is the equilibrium concentration. The linear form of Eq. (3) was obtained by plotting LogC against LogX. The slope and intercept were taken as 1/n and Kf respectively. Phosphorus sorption curves were drawn by plotting the quantity of sorbed P against the P concentration in the equilibrated soil solution Fox [19].

The external P-requirement of each soil or the amount of P required for each soil at 0.2 mg/L equilibrium solution of P also known as the standard P requirement (SPR) was calculated based on Langmuir and Freundlich models/Equations developed for each soil. The soil solution P of 0.2 mg/L was the amount of P that should be available in the soil for optimum plant growth [20].

### 2.7 Statistical data analysis

**A**nalysis of variance was carried out using statics 8 (the latest available version). Mean separation was done using the Least Significant Difference (LSD) test at 5% probability level. In addition, descriptive statistics, and correlation analysis was employed.

## 3 Results and discussions

### 3.1 Soil psysico-chemical properties under different land-use types

Sand, silt, and clay fractions were significantly (p ≤ 0.05) different among the four land uses (Table 1). Selective removal of clay and silt fraction downward through erosion and percolation on cultivated land may result in a higher proportion of sand particles. This is in agreement with the previous finding of Muche et al.*,* [21] in the Farta area, northern Ethiopia. Land-use types had a significant (p 0.05) effect on pH (H_2_O) values (Table 2). The highest soil pH was found on enset land, followed by lime-treated cultivated land, and the lowest was found on grazing and exclosure land-use soils (Table 2). The higher soil pH on enset land use may be due in part to the presence of more total exchangeable bases and PBS than on cultivated land use. Jones [22] classified soil pH values between 5.1 and 5.5 as strongly acidic, pH values between 5.6 and 6.0 as moderately acidic, pH values between 6.1 and 6.5 as slightly acidic, and pH values between 6.6 and 7.3 as neutral. Generally, the pH values recorded for soils of the study area were within the range of neutral for enset while strongly acidic for the other three land uses [23].

**Table 1:** Soil Physical Properties under different land-use types.

LUT	PD	BD	Sand	Silt	Clay	Texture
	gm/cm^3^		%			
Enset	2.26	1.06^c^	33.66^d^	16.00^d^	50.66^a^	Clay
Cultivated	2.47	1.15^b^	54.33^a^	25.33^b^	20.33^c^	Sandy Clay Loam
Grazing Land	2.41	1.23^a^	35.33^c^	44.66^b^	20.33^c^	Loam
Exclosure Area	2.39	1.12^b^	50.60^b^	23.33^c^	25.66^b^	Sandy Clay Loam

**Table 2:** Chemical properties of soil as affected by land-use types at Kuto Sorpela Keble

LUT	pH	Ex.Aci.	Ex. Al	OM	OC	TN	Fe	Av.p	CEC	Ca	Mg
		meq/100 g		%			mg/kg		cmo_l_ (+)kg^−1^		
Enset	6.81^a^	0.45^c^	0.42^d^	3.82^a^	2.21^a^	0.19^a^	1.07^d^	26.49^a^	24.36^a^	6.71^a^	3.06^a^
Cultivated	5.16^b^	1.77^b^	0.81^c^	2.35^d^	1.36^d^	0.12^d^	24.76^c^	14.98^b^	25.16^a^	3.36^b^	1.86^c^
Grazing	5.02^c^	1.96^a^	0.83^b^	3.04^c^	1.76^c^	0.15^c^	27.56^a^	13.95^c^	21.63^b^	2.93^c^	2.13^b^
En. Area	5.02^c^	1.96^a^	1.19^a^	3.48^b^	1.97^b^	0.17^b^	26.36^b^	14.03^c^	24.73^a^	2.93^c^	1.93^c^
LSD_(0.05)_	0.01	0.14	0.01	0.05	0.05	0.006	1.005	0.21	1.14	0.29	0.26
CV (%)	0.98	0.39	1.07	0.92	0.91	1.89	2.52	0.59	2.38	3.75	5.79

LUT = land use types, En. Area = enclosure area, Av.P = available phosphorous, pH = power hydrogen, Ex.Al = exchangeable aluminum, Ex. acidity = exchangeable acidity, OM = organic matter.

Land use types had a significant (P 0.05) effect on organic matter content (Table 2). The soil OM content was highest in enset soil, followed by enclosure area soil, grazing land, and cultivated land (Table 2). Low organic matter input combined with reduced physical protection for SOM as a result of tillage and increased oxidation of soil organic matter could explain the low OM in cultivated land [24].

An analysis of variance revealed that different land use types had a significant (P 0.05) influence on total nitrogen (TN) (Table 2). Enset land had the highest mean value of TN, followed by the enclosure area, and grassland had the lowest value (Table 2). The higher TN for enset land could be attributed to a higher organic matter content. Brady [25] reported that the distribution of soil nitrogen mirrored that of soil OM because nitrogen, like other nutrients, is present in an organic combination and is slowly released by the mineralization process. The available phosphorus (AP) content varied significantly across land-use types (Table 2). The highest AP value was discovered in enset land, followed by grazing land and cultivated soils. The higher available P content on enset land than grazing land was most likely due to long-term manure, house refusal applications such as wood ash, and the resulting increase in microbial activity. According to Landon’s rating [23] the amount of AP in the study area was high for enset, low for cultivated, very low for grass, and enclosure area.

CEC values of the soils in the study area were significantly affected by land use types (Table 2). CEC of soil depends on the relative amounts and types of colloidal substances (organic matter and clay) as both provide negatively charged surfaces that play important role in the exchange process. According to Hazelton and Murphy [26], the topsoils having CEC of >25, 12–25 cmol/kg, 6–12 cmol/kg and <6 cmol/kg are classified as high, moderate, low, and very low, respectively. The soil Ex. Al values were significantly affected by land-use types (Table 2). The highest soil exchangeable Ex. Al was recorded for the soil of the enclosure area followed by grazing land and the lowest was recorded for the soil of the enset (Table 2). The relatively higher soil Ex. Al recorded from the enclosure area might be due to the presence of higher total exchangeable acid and percent acid saturation (Table 2) than that of the enset and cultivated lands. In general, exchangeable aluminum increases and aluminum toxicity may occur below pH 5.5, and phosphorus fixations by Fe and Al increase [27]. The exchangeable calcium (Ca) content of soils of the study area showed significant differences in response to variations in land uses. The high exchangeable Ca was recorded for the surface soil of the enset, whereas the lowest exchangeable Ca was recorded for the enclosure area and grazing land (Table 2).

### 3.2 Phosphorus sorption characteristics

With an increase in the level of added P in the solution, the amounts of P absorbed by the soils under the five types of land use increased. When P was added at any level, the soils of the area enclosure absorbed most of it, followed by grazing land use, untreated cultivated land, lime-treated cultivated land, and enset land use. The Freundlich and Langmuir equations were used to plot the P sorption data for all soil types. For all land uses, the data demonstrated satisfactory agreement with the Langmuir equation (R^2^ > 0.94). Since the adjusted R^2^ values for the Langmuir suggested a good model fit, they were 0.92–0.99.

### 3.3 Phosphorus sorption indices

The P sorption data of the soils could be sufficiently described by the Langmuir model (Table 4). This was demonstrated by the fact that the R values derived from the linear equation were greater than those from Freundlich, demonstrating a significant correlation between the amount of P adsorbable by each soil and the corresponding P concentration in the equilibrium solution. Results from the sorption parameters and some of the soil’s physical-chemical characteristics for the four types of land use were shown in (Table 3, Table 4).

**Table 3:** Phosphorus sorption indices in both models.

LUT	P sorption indices LE						P sorption indices FI		
	X_M_	K	EPR	MBC	R^2^	B	Kf	EPR	R^2^
	mg/kg	L.mg/kg	mg/kg	L/kg		L.kg	mg/kg	Mg/kg	
Enset	136^e^	4.36^a^	45.9^e^	592.96^b^	0.94	0.07^b^	1.22^d^	42.2^d^	0.95
Cultivated soil without Lime	620.7^c^	0.76^c^	180.77^c^	471.70^d^	0.96	0.01^e^	1.83^c^	141.11^c^	0.92
Grazing land	694.3^b^	0.83^b^	244.3^b^	576.25^c^	0.94	0.10^a^	2.35^b^	226.22^b^	0.95
Enclosure Area	731.7^a^	1.3^b^	398.7^a^	957.84^a^	0.94	0.017^d^	2.73^a^	323.33^a^	0.99
Cultivated soil with Lime	526.3^d^	0.6^d^	149.8^d^	315.79^e^	0.98	0.02^c^	1.83^c^	142.3^c^	0.93
LSD_(0.05)_	1.37	0.08	7.63	9.71	–	0.00	0.14	9.17	–
CV (%)	0.13	2.72	1.95	0.89	–	1.03	3.78	4.08	–

LE: Langmuir Equation, MBC: maximum buffering capacity, FI: Freundlich Equation, X_M_ Langmuir sorption maximum, k: binding Energy, SPR: standard p Requirement, Kf: Freundlich surface coverage.

**Table 4:** Chemical properties of cultivated soil affected by lime application.

Management Type	pH	Ex acidity	Ex. Al	Ca	Mg	Fe	Av.p
		cmo_l_ (+)kg^−^				mg/kg	
Cultivated soil without lime	5.16^b^	1.77^a^	2.91^a^	3.36^b^	1.93^b^	25.00^a^	18.03^b^
Cultivated soil with Lime	6.57^a^	0.48^b^	0.033^b^	6.89^a^	3.13^a^	3.49^b^	18.9^a^
LSD_(0.05)_	0.9	0.29	0.034	0.18	0.16	0.024	0.092
CV (%)	0.84	0.4	1.15	2.91	5.9	2.21	0.62

**Table 5:** Correlations of selected soil properties and adsorption capacity.

	CLAY	EPRF	EPRL	K	Kf	OM	pH	X_M_
**EPRF**	−0.5889**							
**EPRL**	−0.5591	0.9806**						
**K**	0.9942**	−0.5621	−0.5300					
**Kf**	−0.6258*	0.9826	0.9555**	−0.5970*				
**OM**	0.6578*	−0.0067	−0.0695**	0.6973	−0.0175*			
**Ph**	0.6578*	−0.6876	−0.7519**	0.4391	−0.6998	0.0646**		
**Clay**	0.0159*	−0.0005	0.0074**	0.0154	−0.0146	0.1049	0.0032**	
**X**_**M**_	−0.8942*	0.8468	0.8459	−0.8634*	0.8665	−0.3993	−0.7937	0.008*

** = highly Significant at 0.05 probability level and * = Significant at 0.05 probability level. All figures in Table 5 without a star (*) symbol are statistically non-significant. X_M_ = Langmuir adsorption maximum, K = binding energy, n and Kf = constant of Freundlich equation, EPRf.

### 3.4 P sorption indices of Langmuir Equation (LE)

Regression coefficients of isotherms in land use and liming showed significant differences (Table 3). The R^2^ of Langmuir was varying from 0.94 to 0.98, while it was between 0.92 and 0.99 for Freundlich. All land-use types had a significant impact on the soil’s maximum sorption values (p 0.05). (Table 3). The observed differences in sorption maxima between soils from different land use systems were most likely caused by differences in the amounts and nature of Al and Fe components present in the soils (Table 4). The lower sorption from enset land use may be due to higher SOM. As a result of the negative correlation between SOM and sorption maximum, it has the potential to increase P sorption. All land-use types had a significant impact on the soil’s sorption binding energy (k) values (Table 3). All of the soils had K values greater than 0.07 Lmg^−1^, indicating that there is no risk of P loss into the water [28]. The amount of energy absorbed varies depending on the land use. The maximum P buffering capacity (MPBC) is a function of k and the Langmuir model XM. This is a capacity factor that measures the soil’s ability to replenish phosphate ions in low concentrations in soil solution, which are often depleted [4].

The external P requirement, also known as SPR, is the amount of P that must be added to the soil to maintain a soil concentration P at 0.2 mg P L^−1^ [20]. A soil solution concentration of 0.2 mg P L^−1^ is the critical concentration of P below which crops/plants will suffer from P deficiency [29]. Soil EPR was significantly affected by land-use types (Table 3). The amount of P required for maintaining a soil solution concentration of 0.2 mg P L^−1^(P_0.2_) ranged from 45.9 to 398.6 mg PL^−1^ soil (Table 3). This indicates that fields need 45.9, 149.8, 180.77, 244.3, 398.7 mg P kg^−1^ soil which is equivalent to 48.6, 172.2, 207.8, 300.4, and 446.5 kg P ha^−1^ or 111.4, 394.1, 476, 688, and 1022.5 kg P_2_O_5_ ha^−1^ to be available for crop growth under enset, lime treated soil, cultivated land without, grazing, and enclosure area respectively. The amount in the form of NPSB (18.9-37.7-6.95-0.1) fertilizer is analogous to 295.4, 1045, 1262.7, 1825.1, and 2712.3 kg P ha^−1^ NPSB for enset, lime treated soil, cultivated soil without, grazing, and enclosure area, respectively whereas, in terms of DAP (0-46-0), it was 242.1, 856.5,1034.9,1495.5 and 2222.9 kg/ha^−1^ for enset, cultivated soil with lime, cultivated soil without, grazing, and enclosure area respectively.

### 3.5 Effects of liming on soil chemical properties of agricultural soil

After applying lime for a month, the results shown in Table 3 showed a significant difference in the measured soil parameters. Liming increased the exchangeable bases (Ca and Mg), available P., and soil pH from 5.17 to 6.5 while decreasing the exchangeable bases (Al and Fe). Because Ca^2+^ ions are likely to displace Al^3+^, H^+^, and Fe^3+^ ions, lime raised the pH of the soil. When H^+^ is neutralized, the pH rises and the proportion of basic cations adsorbed onto soil particles and in solution changes [30]. Al (OH)_3_ and Fe(OH)_3_ are examples of the sesquioxides that are formed when Al and Fe ions that are held by negative sites on soil particle surfaces are displaced into soil solution [30]. The Ca^2+^ could either come from lime or be released upon dissolution of Ca (H_2_PO_4_)_2_. Ca^2+^ could be produced by lime or released when calcium dissolves (H_2_PO_4_)_2_. The reduction of Fe^3+^ and Al^3+^ in solution as a result of Ca^2+^ displacement is caused by the formation of Al (OH_3_) and Fe (OH_3_), which increases P availability in the exchange complex [30].

The ability of soils to adsorb P was significantly impacted by the application of lime. The addition of lime to acidic soils may also raise the pH of the soil, which will facilitate the release of phosphate ions fixed by Al^3+^, H^+^, and Fe^3+^ ions into the soil solution and possibly cause Ca^2+^ ions present in the soil to displace Al^3+^, H^+^, and Fe^3+^ ions. This caused the decrease in P sorption that was seen at all the sites. Similar studies have reported that lime application increases soil pH, available P, lowers Al levels, and increases P sorption in acid soils [5]. Overall, the findings of the present study demonstrated that adding lime to cultivated soil decreased XM P adsorption.

## 4 Conclusion

The purpose of the study was to comprehend the P sorption properties of various land use types and the effects of lime to reclaim cultivated land. In the enclosure area > grazing land-use > cultivated land without lime > cultivated with lime > enset land use of the soils, the results showed significant variation in soil P sorption among different land use types. The maximum amount of P adsorption decreased with the lime application, having a significant impact on soil P adsorption capacity. It was discovered that the Langmuir model accurately described P-sorption. For Enset and the enclosure area, respectively, the P fixation ranged from 136,527–731.67 mg kg^−1^ and the EPR values recorded were between 45.9 and 398.7 mg P kg^−1^ soil. The equivalent amounts for Enset and enclosure area are 295.4 and 2712.3 kg P ha^−1^ NPSB, respectively. Testing model output under greenhouse and field experiments using different crops is advised for practical application and sustainable management. Furthermore, the rate of P applied currently on the traditionally farmed land is low compared to the EPR estimated from the Langmuir equation for each land use in this study. Therefore, it is necessary to take into account the various land-use types when revising the fertilizer recommendation.

## Author contribution statement

Mr. Eshetu: Conceived and designed the experiments; Performed the experiments; contributed reagents, materials, analysis tools or data; wrote the paper.

Alemayehu: Conceived and designed the experiments; Performed the experiments; Analyzed and interpreted the data; wrote the paper.

Fanuel: Conceived and designed the experiments; Performed the experiments; Analyzed and interpreted the data; wrote the paper.

## Funding statement

This research did not receive any specific grant from funding agencies in the public, commercial, or not-for-profit sectors.

## Data availability statement

Data included in article/supp. material/referenced in article.

## Declaration of interest’s statement

The authors declare no competing interests.

## Additional information

No additional information is available for this paper.
